# SHP2 associates with nuclear localization of STAT3: significance in progression and prognosis of colorectal cancer

**DOI:** 10.1038/s41598-017-17604-7

**Published:** 2017-12-14

**Authors:** Yan Huang, Jie Wang, Fuao Cao, Hailong Jiang, An Li, Jianzhong Li, Lei Qiu, Hao Shen, Wenjun Chang, Chuanxiang Zhou, Yamin Pan, Yiming Lu

**Affiliations:** 1 Department of Biochemical Pharmacy, School of Pharmacy, Second Military Medical University, Shanghai, 200433 China; 2 Department of colorectal surgery, Changhai Hospital, Second Military Medical University, Shanghai, 200433 China; 3 Department of Environmental Hygiene, Second Military Medical University, Shanghai, 200433 China; 4 Department of Oral Pathology, Peking University School and Hospital of Stomatology, Beijing, 100081 China; 5 Department of Digestive Endoscopy, Shuguang Hospital, Shanghai University of Traditional Chinese Medicine, Shanghai, 201203 China

## Abstract

Tyrosine phosphatase SHP2, encoded by PTPN11, has been implicated in many physiologic and pathologic processes in neoplastic progression. However, controversies are emerging from many studies, indicating SHP2 has a dual role in different types of tumors. We aimed to explore the role of SHP2 in progression and prognosis of colorectal cancer (CRC). SHP2 inhibited CRC cell proliferation and migration, and the phosphorylation of STAT3 was negatively regulated by SHP2 in CRC. SHP2 and nuclear STAT3 were examined in 270 CRC tissues. SHP2 was significantly correlated with nuclear STAT3 (Spearman’s rho = −0.408, P ≤ 0.001). Based on Cox regression analysis, patients with high levels of SHP2 and low levels of nuclear STAT3 had longer disease-specific survival (DSS) (HR, 0.362; 95% CI, 0.165–0.794) and disease-free survival (DFS) (HR, 0.447; 95% CI, 0.227–0.877). Further, low levels of SHP2 and high levels of nuclear STAT3 were independently associated with adverse outcomes in the whole cohort (DFS; HR, 2.353; 95% CI, 1.199–4.619). These results suggest that combination of SHP2 and nuclear STAT3 is a strong prognostic predictor in CRC.

## Introduction

Colorectal cancer (CRC) is the third most frequent cancer in men and second in women worldwide^1^. Surgical resection remains the most effective treatment for patients with localized and regional carcinoma^2^. However, a subset of patients will develop recurrence and metachronous metastases after surgical resection, and recurrence is the main cause of death^3^. Post-operative chemotherapy improves the clinical outcomes, including disease-specific survival (DSS) and disease-free survival (DFS), and reduces tumor recurrence rates, but overtreatment is harmful, owing to the side effects of chemotherapy^4^. Therefore, it is of great importance to develop certain selective criteria to properly address post-operative surveillance and treatments.

The Src homology-2 domain-containing phosphatase, SHP2, encoded by *PTPN11*, is composed of a single phosphor-tyrosine phosphatase (PTP) domain and two N-terminal SH2 domains. The C-terminal region of SHP2 contains sites of tyrosine phosphorylation and a proline-rich region^5^. In most, if not all conditions, SHP2 is required to activate the RAS-ERK pathway^6,7^. However, depending on the cell or tissue type, SHP2 also enhanced or antagonized PI3K-AKT or RHO activation^8,9^, increased STAT5 phosphorylation^10^, but decreased STAT3 phosphorylation^11^, and might affect NF-кB^12^ or NFAT^13^ pathways. Such a complexity makes it difficult to identify SHP2 functions.

Recent studies show that ablation of SHP2 in the intestine epithelial cells (IECs) results in the spontaneous development of colitis^14–16^. Genetic studies identified SHP2-encoding *PTPN11* as an inflammatory bowel diseases (IBDs) susceptibility gene, and SHP2 is associated with intronic polymorphismas described in Japanese patients with ulcerative colitis (UC)^17^. Coulombe *et al*. demonstrated that SHP2 transcripts are significantly reduced in patients with UC, implying that SHP2 expression is affected by *PTPN11* intronic polymorphisms^15^. Based on the close connection between inflammation and CRC, SHP2 may be a key molecule in the progression of CRC. We previously showed that SHP2 is weakly expressed in epithelial cells of adenoma and CRC specimens, and high SHP2 expression in CRC is associated with good prognosis, indicating that SHP2 tends to be a tumor suppressor in CRC^18^. However, current studies do not provide insights into the biological mechanisms underlying the clinical behavior of SHP2, and exploratory studies combined with SHP2 correlated regulators or downstream targets should be performed to further discriminate CRC patients with favorable or unfavorable outcomes. The present study explored the role of SHP2 on the aggressiveness of CRC cells through knockdown, overexpression, and functional inhibition. Moreover, we investigated the correlation between SHP2 and JAK2/STAT3 signaling *in vitro* and in tumor tissues from patients with CRC. Further, we evaluated the prognostic value of the combination of SHP2 and STAT3 in tumor samples from a cohort of CRC patients with known clinical history.

## Results

### SHP2 inhibits CRC cell proliferation and migration

To explore the role of SHP2 in CRC progression, SHP2 expression levels in different CRC cells were determined by western blot. SHP2 was highly expressed in HCT116 and RKO cells, but was relatively low in LoVo and SW480 cells (Supplementary Fig. S1A).

**Figure 1:**
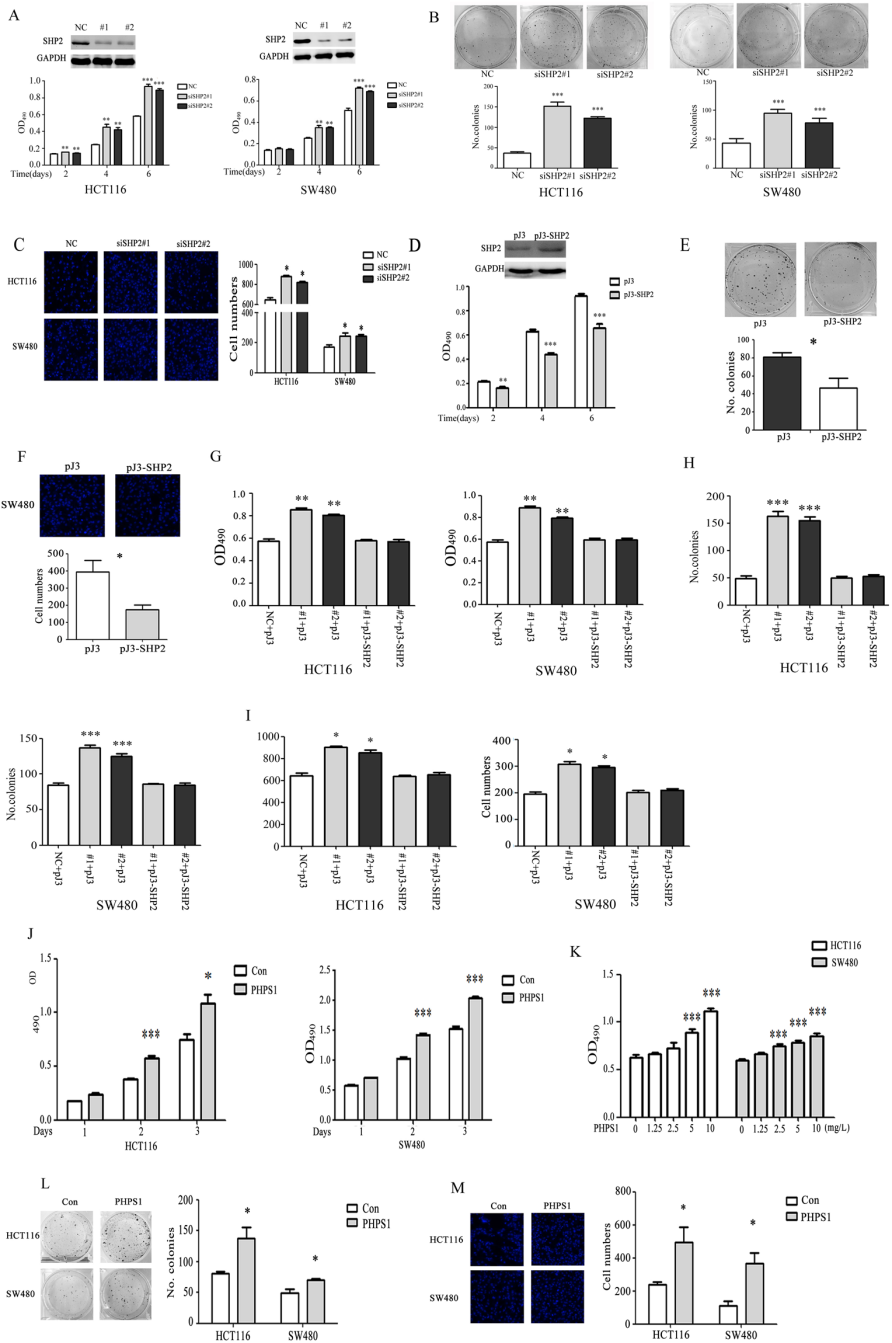
SHP2 inhibits CRC cell proliferation and migration. (**A**) SHP2 knockdown by siSHP2#1 and #2 markedly increased the proliferation of HCT116 and SW480 cells. (**B**) Colony formation assays were conducted to estimate the growth rate of HCT116 and SW480 cells. SHP2 knockdown increased the colony numbers compared with the control group. Representative pictures of colonies (left) and quantification of colony numbers (right) are shown. (**C**) Transwell assay was performed to access the effect of SHP2 on cell migration by siRNA mediated knockdown. Representative pictures of cells (left) and quantification of cell numbers (right) are shown. (**D**) SW480 cells were transfected with pJ-SHP2 plasmid to overexpress SHP2 and the cell proliferation was accessed by MTT assay. (**E**) Colony formation assay was done by overexpression of SHP2 in SW480 cells. SHP2 overexpression decreased the colony numbers compared with the control group. Representative pictures of colonies (left) and quantification of colony numbers (right) are shown. (**F**) Transwell assay was performed to access the effect of SHP2 on cell migration by overexpressing SHP2 in SW480 cells. Representative pictures of cells (left) and quantification of cell numbers (right) are shown. Overexpression of SHP2 in HCT116 and SW480 cells rescued SHP2 knockdown induced cell proliferation (**G**), colony formation (**H**) and cell migration (**I**). (**J** and **K**) PHPS1 improved HCT116 and SW480 cell proliferation during 3 days in a time- and dose-dependent manner. (**L**) Cells were treated with PHPS1, and colony formation was measured after two weeks by crystal violet staining. PHPS1 increased the number of CRC cell colonies. (M)Transwell assay showing blockade of SHP2 phosphatase activity by PHPS1 improved CRC cell migratory ability. NC, non-silencing control siRNA. Values represent mean ± SEM (n = 3–4), *P ≤ 0.05, **P ≤ 0.01, ***P ≤ 0.001.

To elucidate the effect of decreased SHP2 on CRC cell proliferation and migration, HCT116 and SW480 cells were transfected with two sets of SHP2-specific siRNA (#1 and #2). As shown in Fig. 1A,B and C, SHP2 knockdown significantly promoted the proliferation, colony formation and migration of HCT116 and SW480 cells. Meanwhile, SHP2 overexpression significantly inhibited the proliferation, colony formation and migration of SW480 cells (Fig. 1D,E and F). To exclude the possible off-target effects and further identify SHP2 function, SHP2 plasmid was transiently overexpressed in HCT116 cells and SW480 cells after SHP2 siRNA (#1 and #2) mediated knockdown. As shown in Supplementary Fig. S1B and Fig. 1G–I, SHP2 overexpression could rescue the effects of siRNA mediated knockdown on protein expression and cell proliferation, colony formation and migration.

To check whether SHP2 effects in CRC cells are due to its phosphatase activity, PHPS1, an inhibitor of SHP2 phosphatase activity^19^, was introduced. As shown in Fig. 1J,K and L, blockage of SHP2 catalytic activity by PHPS1 improved the proliferation of CRC cells in a dose- and time-dependent manner and colony formation in 2D cell culture condition. Meanwhile, PHPS1 promoted the migration of CRC cells compared with the control group (Fig. 1M).

Collectively, these data indicated that SHP2, with its catalytic activity, inhibited CRC cell proliferation and migration.

### SHP2 inhibiting CRC cell proliferation via STAT3 dephosphorylation

**Figure 2:**
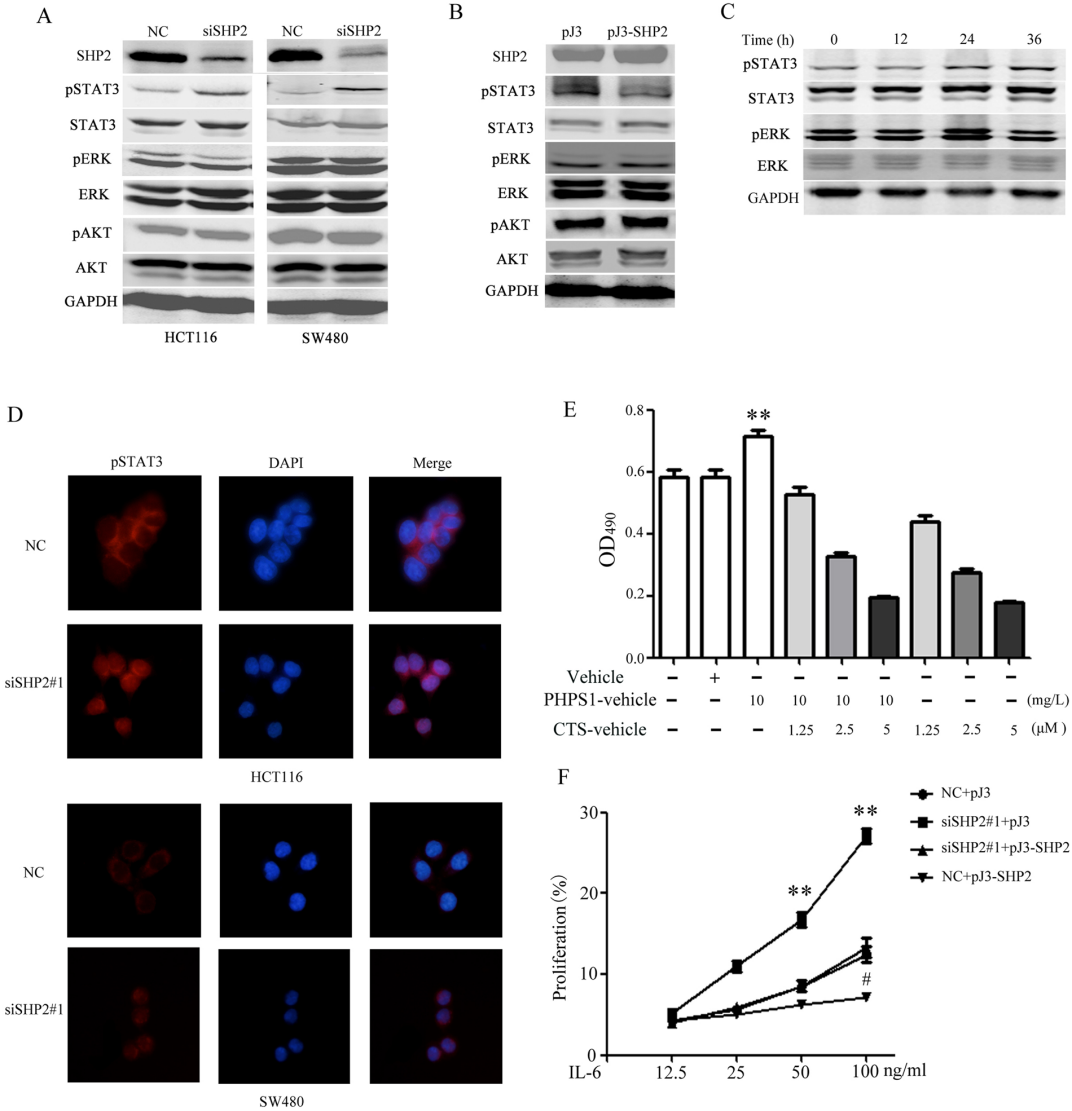
SHP2 suppressing role in CRC is mediated by STAT3 dephosphorylation. (**A**) STAT3 phosphorylation was increased in CRC cells knocked down for SHP2 by siRNA#1 and #2. (**B**) SHP2 overexpression reduced STAT3 phosphorylation in SW480. (**C**) PHPS1 enhanced the levels of pSTAT3 in 10 mg/L time-dependently. (**D**) Nuclear distribution of pSTAT3 was enhanced after SHP2 knockdown (×400). (**E**) Cryptotanshinone significantly reversed CRC cell proliferation induced by PHPS1. Values represent mean ± SEM (n = 3), **P ≤ 0.01, compared with Vehicle group. (**F**) SW480 was more sensitive to IL-6-induced proliferation after SHP2 knockdown, which was rescued by SHP2 overexpression; meanwhile overexpression SHP2 inhibited IL-6 induced SW480 proliferation. Values represent mean ± SEM (n = 3) ^#^P ≤ 0.05, **P ≤ 0.01, compared with NC + pJ3 group. Whole blots are shown in Supplementary Fig. S5.

In probing underlying mechanisms, we analyzed the phosphorylation of ERK1/2, AKT, and STAT3 in CRC cells after transfection with the SHP2 siRNA #1. STAT3 phosphorylation increased upon SHP2 knockdown in CRC cells (Fig. 2A) and, consistently, pSTAT3 was obviously attenuated in SW480 cells overexpressing SHP2 (Fig. 2B). The SHP2 inhibitor, PHPS1 at 10 mg/L, enhanced STAT3 activation (Fig. 2C). As expected, the nuclear localization of pSTAT3 was dramatically enhanced by SHP2 interference in CRC cells (Fig. 2D). Overall, SHP2 inhibited STAT3 phosphorylation and nuclear translocation in CRC cells. Collectively, data from western blot and immunofluorescence implied that pSTAT3 was negatively regulated by SHP2 in CRC cells, which might be a function different from that in other carcinomas such as leukemia^20,21^ and breast cancer^22,23^. Moreover, we performed rescue experiments by inhibiting STAT3 functionally. Cryptotanshinone, which could inhibit STAT3 Tyr705 phosphorylation, was introduced. As shown in Fig. 2E, the proliferation induced by PHPS1 was rescued by Cryptotanshinone (CTS). Overexpression SHP2 inhibited IL-6 induced SW480 proliferation (Fig. 2F). Moreover, siRNA-mediated SHP2 silencing sensitized SW480 cells to IL-6-induced proliferation, which was recused by SHP2 overexpression (Fig. 2F).

### Correlation between SHP2 and nuclear localization of STAT3 in CRC tissues

STAT3 is phosphorylated by receptor-associated kinase and then forms homo-or heterodimers that translocate to the cell nucleus, where they transcribe a variety of genes that are associated with cancer cell growth and metastasis^24^. Therefore, to further identify the possible correlation between SHP2 and pSTAT3, we used tissue microarrays to determine the expression of SHP2 and STAT3 by IHC. Typical immunostainings are shown in Supplementary Fig. S2. SHP2 immunoreactivity was mainly observed in the cytoplasm and nucleus in cancer cells in CRC specimens, and SHP2 was poorly expressed in advanced-stage CRC. STAT3 staining was mainly distributed in the cytoplasm and nucleus in cancer cells, and nuclear localization was increased in advanced-stage CRC. Taking the IHC-score as a continuous variable, we performed the Spearman’s r test to evaluate the correlation between the expression of SHP2 and nuclear STAT3, a significant negative correlation was observed (Spearman’s rho = −0.408, *P* ≤ 0.001).

**Figure 3:**
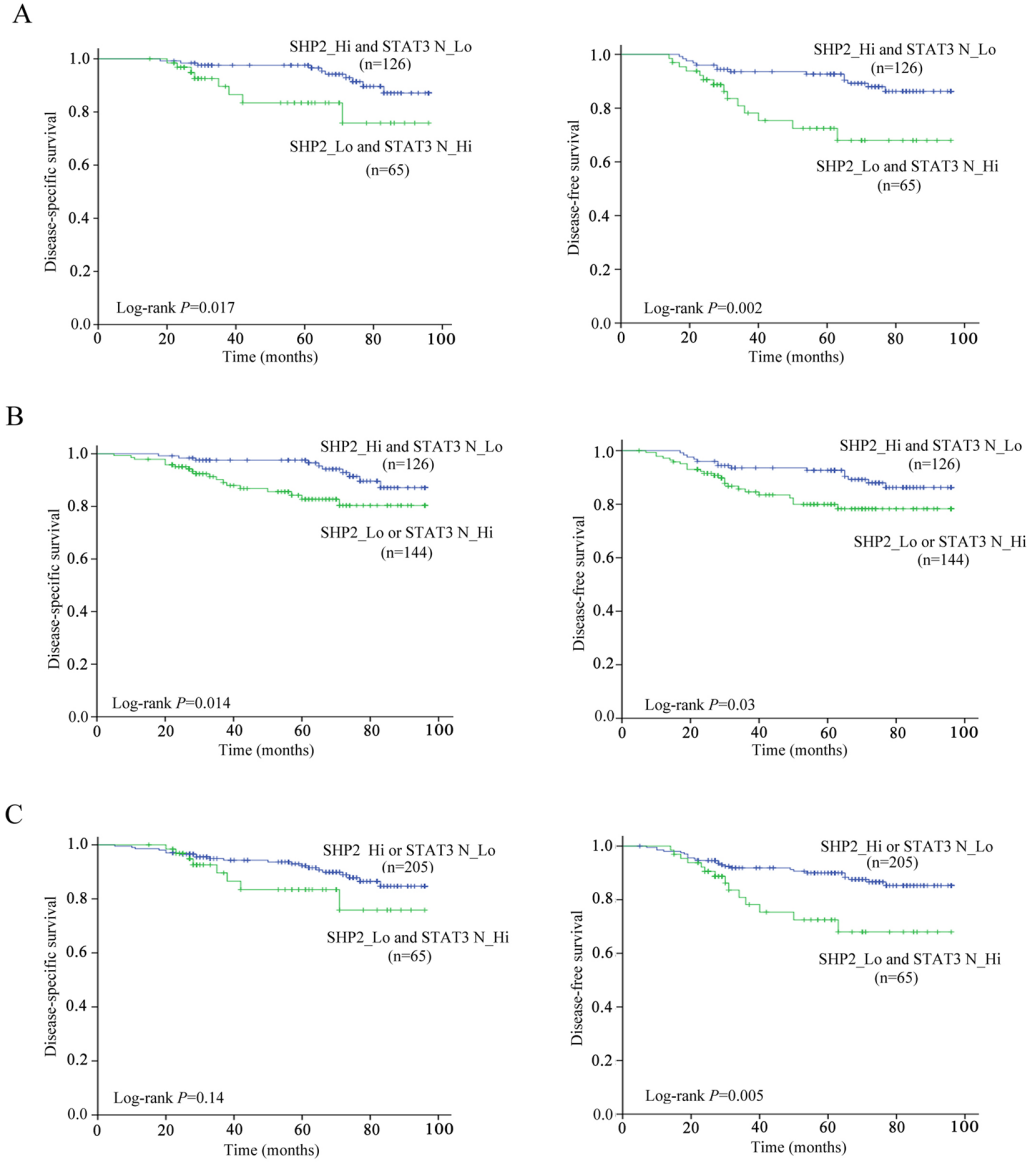
Association between the combination of SHP2/nuclear STAT3 and survival in patients with CRC. (**A**) Kaplan–Meier analysis showed that Patients with high SHP2 and low nuclear STAT3 present better DSS (left panel) and DFS (right panel) than patients with low SHP2 and high nuclear STAT3. (**B**) Kaplan-Meier analysis showed that Patients with high SHP2 and low nuclear STAT3 present better DSS (left panel) and DFS (right panel) than patients with low SHP2 or high nuclear STAT3. (**C**) Kaplan-Meier analysis showed that patients with high SHP2 or low nuclear STAT3 present better DFS (right panel) but not DSS (left panel) than patients with low SHP2 and high nuclear STAT3. P values were determined using log-rank test.

Moreover, Kaplan-Meier survival analysis showed that high levels of SHP2 and low levels of nuclear STAT3 (SHP2_Hi and STAT3_Lo) in primary tumors (n = 126) were significantly associated with better DSS (P = 0.017) and DFS (P = 0.002) compared with low levels of SHP2 and high levels of nuclear STAT3 (SHP2_Lo and STAT3_Hi) in primary tumors (n = 65) (Fig. 3A).

### Prognostic values of the combination of SHP2 and nuclear STAT3

To further assess the potential prognostic values of the combination of SHP2 and nuclear STAT3 expression, we firstly dichotomized the patients into 2 subgroups (SHP2_Hi and STAT3_Lo *vs*. others) according to their status of SHP2 and nuclear STAT3 levels. Kaplan-Meier survival analysis showed that patients with high levels of SHP2 and low levels of nuclear STAT3 (SHP2_Hi and STAT3_Lo) were significantly correlated with better DSS (*P = *0.014) and DFS (*P = *0.03) compared with other patients (SHP2_Lo or STAT3_Hi) (Fig. 3B). Distribution of demographic and clinical parameters such as gender, disease location, differentiation degrade, TNM, T stage, N stage, adjuvant chemotherapy, serum CEA, and CA19–9 did not vary significantly between the two subgroups (Supplementary Table S1). Except for SHP2_Hi and STAT3_Lo (HR, 0.388; 95% CI, 0.178–0.842) and serum CEA, other factors such as TNM and serum CA19-9 were not significantly associated with DSS in univariate Cox analysis. SHP2_Hi and STAT3_Lo (HR, 0.481; 95% CI, 0.247–0.936) and gender were significantly associated with DFS in univariate Cox analysis (Table 1). In multivariate Cox analysis, SHP2_Hi and STAT3_Lo (HR, 0.362; 95% CI, 0.165–0.794) and serum CEA remained independent predictors for DSS, while SHP2_Hi and STAT3_Lo (HR, 0.447; 95% CI, 0.227–0.877) and gender were independently associated with DFS (Table 2).

**Table 1:** Univariate Cox regression analysis for clinical parameters and the combinations of SHP2 and nuclear STAT3.

Variables	DSS		DFS	
	HR (95% CI)	*P*-value	HR (95% CI)	*P*-value
SHP2_Hi and STAT3_Lo *vs*. others	**0.388 (0.178–0.842)**	**0.017**	**0.481 (0.247–0.936)**	**0.031**
SHP2_Lo and STAT3_Hi *vs*. others	1.781 (0.780–4.066)	0.171	**2.443 (1.253–4.763)**	**0.009**
Age (>50 *vs*. ≤50)	1.388 (0.483–3.989)	0.543	0.713 (0.337–1.506)	0.375
TNM (III *vs*. I + II)	1.551 (0.749–3.227)	0.24	1.62 (0.854–3.071)	0.139
Sex (women *vs*. men)	1.892 (0.856–4.164)	0.113	**2.282 (1.107–4.704)**	**0.025**
Disease location (colon *vs*. rectum)	1.032 (0.479–2.225)	0.936	0.984 (0.508–1.905)	0.961
Adjuvant chemotherapy (yes *vs*. no)	1.185 (0.452–3.109)	0.730	2.162 (0.767–6.093)	0.145
Serum CEA (ng/mL) (≥5 *vs*. < 5)	**2.433 (1.160–5.101)**	**0.019**	1.841 (0.974–3.482)	0.60
Serum CA19-9 (U/mL) (≥37 *vs*. <37)	1.869 (0.797–4.384)	0.151	1.273 (0.560–2.894)	0.564

**Table 2:** Multivariate Cox regression analysis for clinical parameters and the combinations of high SHP2 and low nuclear STAT3.

Variables	DSS		DFS	
	HR (95% CI)	*P*-value	HR (95% CI)	*P*-value
SHP2_Hi and STAT3_Lo vs SHP2_Lo or STAT3_Hi	**0.362 (0.165–0.794)**	**0.011**	**0.447 (0.227–0.877)**	**0.019**
Age (>50 *vs* ≤50)	1.296 (0.44–3.811)	0.638	0.684 (0.317–1.476)	0.333
TNM (III *vs* I + II)	1.445 (0.638–3.273)	0.377	1.414 (0.708–2.825)	0.327
Sex (women *vs* men)	0.453 (0.203–1.013)	0.054	**0.393 (0.187–0.824)**	**0.013**
Disease location (colon*vs*rectum)	1.010 (0.464–2.202)	0.980	0.922 (0.468–1.817)	0.814
Adjuvant chemotherapy (yes *vs* no)	0.985 (0.34–2.858)	0.978	1.769 (0.596–5.252)	0.304
Serum CEA (ng/mL) (≥5 *vs* <5)	**2.221 (1.021–4.831)**	**0.044**	1.919 (0.977–3.767)	0.058
Serum CA19-9 (U/mL) (≥37 *vs* <37)	1.528 (0.615–3.798)	0.362	1.073 (0.445–2.591)	0.875

We then dichotomized the same cohort into 2 other subgroups (SHP2_Lo and STAT3_Hi *vs*. others). Kaplan-Meier survival analysis confirmed that the presence of SHP2_Lo and STAT3_Hi was significantly correlated with worse DFS (P = 0.005), but not with DSS (P = 0.14) (Fig. 3C). Clinical parameters did not vary significantly between the two subgroups (Supplementary Table S1). In univariate Cox analysis, SHP2_Lo and STAT3_Hi (HR, 2.443; 95% CI, 1.253–4.763) strongly predicted the prognosis of worse DFS (Table 1). Moreover, multivariate Cox analysis demonstrated that SHP2_Lo and STAT3_Hi (HR, 2.353; 95% CI, 1.199–4.619) remained an independent prognostic factor for DFS (Table 3).

**Table 3:** Multivariate Cox regression analysis of DFS for clinical parameters and the combinations of low SHP2 and high nuclear STAT3.

Variables	HR (95% CI)	*P*-value
SHP2_Lo and STAT3_Hi vs SHP2_Hi or STAT3_Lo	**2.353 (1.199–4.619)**	**0.013**
Age (>50 *vs* ≤50)	0.701 (0.326–1.507)	0.363
TNM (III *vs* I + II)	1.347 (0.673–2.694)	0.400
Sex (women *vs* men)	**0.410 (0.197–0.854)**	**0.017**
Disease location (colon*vs* rectum)	0.863 (0.435–1.713)	0.674
Adjuvant chemotherapy (yes *vs* no)	1.807 (0.611–5.345)	0.285
Serum CEA (ng/ml) (≥5 *vs* <5)	1.806 (0.921–3.541)	0.085
Serum CA19-9 (U/mL) (≥37 *vs* <37)	1.189 (0.498–2.840)	0.696

Additionally, the prognostic value of single SHP2 or STAT3 was evaluated by Kaplan-Meier survival analysis and Cox regression analysis, respectively. As shown in Supplementary Table S2 and Fig. S3, the IHC score of SHP2 showed no association with DSS and DFS. Nuclear STAT3 levels were independently associated with DSS and DFS in Cox univariate analysis. However, in multivariate Cox analysis, nuclear STAT3 levels were not independently associated with DSS and DFS after adjusting for variables (Supplementary Table S2), which emphasized the prognostic values of the combination of the levels of SHP2 and nuclear STAT3.

## Discussion

In this study, we demonstrated that SHP2 reduced the aggressiveness of CRC cells *in vitro*, and had a robust capability of inhibiting STAT3 activation in CRC cells. Tissue microarray analysis indicated that SHP2 levels significantly and negatively correlated with nuclear STAT3 levels. Furthermore, combination of the SHP2 status and nuclear STAT3 levels was used to dichotomize patients with CRC into different subgroups with significantly different risks of death and outcomes.

A series of studies demonstrated that deregulation of the SHP2-RAS-ERK signaling cascade is a common pathogenic feature in a number of solid and hematologic malignancies such as breast cancer, gastric cancer, and juvenile myelomonocytic leukemia (JMML)^22,23,25–27^. However, currently, many studies demonstrated that SHP2 acts as a tumor suppressor in numerous carcinomas. Bard-Chapeau *et al*. demonstrated that mice with hepatocyte-specific deletion of SHP2 are more sensitive to diethylnitrosamine (DEN)-induced hepatocellular carcinoma (HCC), and STAT3 is required for the promotion of HCC development induced by SHP2 deletion^11^. Furthermore, in HCC cells, SHP2 inhibits JAK2/STAT3 activation induced by growth arrest and DNA damage 45 G (GADD45G) expression, which inhibits HCC by inducing cellular senescence^28^. Moreover, sorafenib inhibits STAT3 signaling in cholangiocarcinoma cells (CCA) by activating SHP2^29^. Similar to CCA and HCC, CRC is often raised from an inflammatory environment^30,31^. Inflammation-associated carcinogenesis is partly mediated by deregulation of cytokine signaling pathways^32^, which could potentially be therapeutic targets and prognostic markers. Tyr705phospho-STAT3 could be deregulated in some gastrointestinal tumors^33,34^. Qi *et al*. demonstrated the important role of SHP2 in carcinoma progression, indicating SHP2 inhibits proliferation of esophageal squamous cell cancer via dephosphorylation of STAT3^35^. These reports imply that SHP2 plays different roles in different types of cancer via STAT3 dephosphorylation. Currently, few articles focused on the role of SHP2 in CRC. Our current observation indicates that SHP2 could suppress the aggressiveness of CRC cells and, simultaneously, negatively regulate STAT3 activation. The rescue assay confirmed that PHPS1 failed to induce HCT116 proliferation in combination with Cryptotanshinone, compared with the cell treated with PHPS1 only and SHP2 knockdown sensitized SW480 cells to IL-6-induced proliferation, which was rescued by SHP2 overexpression. Together with previous studies^11,28,29^, we now proposed that STAT3 inhibition is required for SHP2 suppressor role in CRC cells.

Prognostic factors and biomarkers are significant in fundamental research and clinical application. Currently, studies examining the correlation between SHP2 or STAT3 with CRC prognosis have been reported^18,36,37^, but no study clearly established the association between the combination of SHP2/nuclear STAT3 status and the outcomes of patients with CRC. In this study, we determined a robust significant correlation between the status of SHP2 and nuclear STAT3 and important clinical outcomes (DFS and DSS) in patients with CRC. To better evaluate the prognostic values of the combination of the two proteins, we compared the outcomes of patients with “SHP2_Hi and STAT3_Lo” or “SHP2_Lo and STAT3_Hi” with these of other patients respectively. The two types of combination efficiently stratified CRC patients with distinct prognosis in the same cohort and exhibited stronger stratification power than the single markers did (Supplementary Table S2), indicating that the discriminating effect of SHP2 and STAT3 in predicting CRC prognosis was complementary.

STAT3, a member of the STAT protein family, is constitutively activated in a series of human carcinomas, including CRC, and plays a crucial role in proliferation, survival, metastasis, and angiogenesis in cancer^24^. Besides, STAT3 is a promising target for the development of clinical therapeutic agents for carcinoma chemoprevention^38–41^. Patients with “SHP2_Lo and STAT3_Hi” were associated with unfavorable prognosis, implying that the predictor could be used to direct postoperative chemotherapy against STAT3, or the JAK2/STAT3 pathway^42–44^, or to develop single-stranded antisense oligonucleotides (ODNs) targeting STAT3^45,46^.

However, the current study presents few limitations. First, patients enrolled in this study were all from a single center, a larger multi-center validation cohort is necessary to verify the clinical value of using the status of SHP2 and STAT3 level as a predictor. Secondly, although primary data of the intratumoral heterogeneity has been assessed, but the effect of intratumoral heterogeneity on the prognosis value of the combination should be systemically evaluated using large cohorts. Thirdly, the current cohort analysis lacked substantial statistical power due to the limited sample size. Finally, some important clinical parameters such as tumor differentiation grade (given the very limited sample size in 1 and 3 grade), vascular, lymphatic and perineural invasion (L, V and P stage), and microsatellite instability were not included, which led to an incomplete inclusion of variants for multivariate Cox regression analysis.

In summary, our study indicates that the combination of SHP2 and nuclear STAT3 levels is a strong prognostic predictor in CRC. Further prospective studies are needed to verify and understand the clinical values of the status of these two markers.

## Methods

### Patients

This study was approved by the institutional review board of Shuguang Hospital and a written informed consent was obtained for each patient. We performed the study on pathologically proven formalin-fixed, paraffin-embedded (FFPE) specimens collected at surgery from 270 patients with stage I–III CRC who underwent surgical resection at Shuguang Hospital, Shanghai University of Traditional Chinese Medicine (Shanghai, China) between June 2001 and February 2009. Clinicopathological parameters of each patient are summarized in Supplementary Table S1. TNM stage was reclassified according to the America Joint Committee on Cancer staging manual (seventh edition). The rule-based post-operative chemotherapy was the FOLFOX regimen. The diagram and search criteria of the study patients in the cohort are presented in Supplementary Fig. S4. All participants are self-reported Han Chinese.

### Cell lines, reagents, and transfection

Human CRC cells, HCT116, RKO, SW480, and LoVo, were obtained from the ATCC (Manassas, VA, USA) and cultured in RPMI-1640 medium (Sigma, St Louis, MO, USA) containing 10% (v/v) fetal bovine serum (FBS, GIBCO, BRL) at 37 °C in 5% CO2. PHPS1 (MW: 465.44) was purchased from Sigma. Cryptotanshinone (MW: 296.36) was purchased from Selleck (SH, China). Both were dissolved in dimethyl sulfoxide (DMSO) for further dilution. Two sets of siRNA targeting SHP2 (siSHP2#1 and siSHP2#2) were designed and used to knockout the expression of SHP2^18,29^. Wild-type SHP2 expressing plasmid pJ3 (pJ3-SHP2 WT) was a gift from Ben Neel (Addgene plasmid # 8317)^47^. Cells were grown to 50% and to 80–90% confluence for siSHP2 and pJ3-SHP2 transfection using Hieff Trans (Yeasen, SH, China), respectively. The effects of gene knockdown and overexpression were assessed by western blot after 48 h.

### MTT assay

After transfection for 12 h, human CRC cells were re-seeded into 96-well plates in 100 μL medium. The cells were incubated separately for 2, 4, and 6 days. MTT solution was added to each well and cells were cultured for another 4 h. The supernatant in the wells was then removed and 150 μL DMSO was added. The optical density (OD) value was determined at 490 nm and OD_490_ was used to represent the number of cells.

To determine the effect of PHPS1, human CRC cells were seeded into 96-well plates. When the cells entered anchorage growth, PHPS1 was added at 1.25, 2.5, 5, and 10 mg/L to test the dose-dependent effect. After 3 days of incubation, MTT was added and OD value was determined. For the time dependent effect, PHPS1 (10 mg/L) was added to the cells at anchorage growth and the OD value was determined after 24, 48, and 72 h.

To assess the effect of SHP2 on IL-6-induced CRC cell proliferation, the same numbers of cells were re-seeded into 96-well plates after transfection 24 h. When cells entered anchorage growth, the medium was replaced by medium without FBS and IL-6 (12.5, 25, 50, and 100 ng/mL) was added. After 2 days of incubation, MTT was added and the OD value was determined.

### Colony formation assay

After transfection for 12 h, human CRC cells were re-seeded into 6-well plates in 2 mL of medium. After 2 weeks of culture, the resulting colonies were fixed with ice-cold methanol and stained with crystal violet solution (Beyotime, Shanghai, China) for counting. For PHPS1, cells were seeded into 6-well plates in 2 mL of medium. After incubation for 24 h, 10 mg/L PHPS1 was added, and the medium containing PHPS1 was renewed every 3 days. After 2 weeks of culture, colonies were stained with crystal violet solution for counting.

### Cell migration assay

Twenty four-well transwell chambers (Corning, Corning, NY, USA) were used to determine the migration of CRC cells. Transfected CRC cells in serum-free medium were re-seeded into the upper chamber at the number of 2 × 10^5^ cells per chamber. The lower chamber was filled with RPMI-1640 medium supplemented with 10% FBS. After 18 h of incubation, migrated cells on the lower chamber were fixed with 4% paraformaldehyde (Sigma), stained with the nuclear probe DAPI (Sigma), and counted.

### Immunofluorescence

After 12 h of transfection, CRC cells were re-seeded into 6-well plates where coverslips coated with poly-L-lysine had been added. After 48 h of incubation, medium was removed and cells were washed with cold PBS. Ice-cold 4% paraformaldehyde was used to fix the cells for 15 min then cells were permeabilized for 5 min with 0.1% Triton X-100 in PBS. The cells were washed with PBS 3 times. Then cells were incubated with the primary antibody diluted in PBS supplemented with 4% horse serum overnight at 4 °C. The following day, the cells were washed 3 times with PBS and then incubated with the secondary antibody for 1 h at room temperature. After antibody incubation, cells were counterstained with the nuclear probe DAPI (Sigma).

### Western blot assay

Cell extracts were resolved in a 10% SDS-PAGE gel and transferred to nitrocellulose filter membrane. The antigens were detected by specific antibodies followed by the respective secondary antibodies (LI-COR Biotechnology, Lincoln, NE, USA). GAPDH was simultaneously detected as a loading control. Detection was performed with an Odyssey infrared imaging system (LI-COR Biotechnology). The following antibodies were used: rabbit monoclonal anti-SHP2, rabbit monoclonal anti-STAT3, and rabbit monoclonal anti-GAPDH (Santa Cruz Biotechnology, Santa Cruz, CA, USA), and rabbit monoclonal anti-ERK, rabbit monoclonal anti-AKT, rabbit monoclonal anti-phospho-ERK, rabbit monoclonal anti-phospho-STAT3 (Tyr705) and rabbit monoclonal anti-phospho-AKT (Ser473) (Cell Signaling Technologies, Beverly, MA, USA).

### Immunohistochemistry

Tissue microarrays containing the FFPE specimens from Shuguang Hospital were commercially developed (Outdo Biotech, Shanghai, China) as previously described^48^. Rabbit monoclonal antibodies to SHP2 (1:100, #sc-280, Santa Cruz Biotechnology) and rabbit monoclonal antibodies to STAT3 (1:100, #sc-482, Santa Cruz Biotechnology) were used for immunohistochemistry (IHC) examination according to the manufacturer’s instructions. Intratumoral heterogeneity of the markers was evaluated by examining five randomly selected spots of each mount from the remaining block of tissue specimens originally used for the development of TMA from 5 patients. Coefficient of variation (CV) was used to assess the intratumoral heterogeneity of IHC separately for each marker as described previously^49^. Due to the skewed distribution of the CV value (higher in high IHC score), so we presented the data of CV for SHP2 and STAT3 amongst the 5 patients by the calculating the median and interquartile range (IQR).The CV (IQR) for SHP2 and STAT3 is 18.5% (11.8–23.1%) and 13.9% (11.0–26.8%). For every case, one tumor punch was stained and the center region was evaluated. A minimum of 100 cells and 3 randomly selected spots were evaluated to calculate the IHC-score. The scores of SHP2 and nuclear STAT3 were based on the intensity of the staining (0 = no staining, 1 = weak staining, 2 = moderate staining, and 3 = strong staining) and percentage of stained cells (0: < 4%; 1: 5–24%; 2: 25–49%; 3: 50–74% and 4: >75%)^18^. Scores for intensity and percentage of stained cells were multiplied for a maximum score of “12”. X-tile software (http://medicine.yale.edu/lab/rimm/research/software.aspx)^50^ was used to determine the optimal cut-off value of SHP2 and nuclear STAT3 expression. The cut-off value of 9 of IHC-score was selected to define patient subgroups with high or low levels of SHP2 (SHP2_Hi or SHP2_Lo); and the cut-off value 4 of IHC-score was selected to dichotomize patients into subgroups with high or low nuclear levels of STAT3 (STAT3_Hi or STAT3_Lo). IHC-scores were independently reviewed for scoring by two observers (AL and YB) who were blinded to the clinical data. The concordance between the scoring results obtained by two independent observers was evaluated with the use of contingency tables and by calculation of Cohen’s kappa indexes as described previously^49^ (Supplementary Tables S3 and S4). The results showed an excellent agreement, both for SHP2 (Κ = 0.785) and STAT3 (Κ = 0.749).

### Follow-up and survival analysis

Follow-up examination was performed as previous described^18^, and the median follow-up period was 68 months (IQR 33–82). Patients with intact clinicopathological parameters were included in the survival analysis. DSS was measured as the time interval from the date of surgery to the date of death from CRC. DFS was defined in months from the date of surgery to the date of first relapse. We dichotomized the entire cohort into 2 coupled subgroups (SHP2_Hi and STAT3_Lo *vs*. others; SHP2_Lo and STAT3_Hi *vs*. others) according to their status of SHP2 and nuclear STAT3 levels, which can effectively discriminate the prognosis differences of patients with CRC.

### Statistical analysis

Spearman’s r test was used to evaluate the correlation between SHP2 and nuclear STAT3 expression. Coefficient of variation (CV) was used to assess the intratumoral heterogeneity of IHC separately for each marker. χ^2^ Test, Student t test, and Mann-Whitney U test were performed to assess the association of the combination of SHP2 and nuclear STAT3 levels with clinicopathological parameters. The Kaplan-Meier method with log-rang test was used to evaluate DSS and DFS. Cox regression analysis was used to estimate the HRs (hazard ratios) and corresponding 95% CIs (confidence intervals). The P values were two-sided for all statistical analyses, and SPSS 21.0 software for Windows (Chicago, IL, USA) was used. *P* ≤ 0.05 was considered statistically significant.

### Statement

All methods were carried out in accordance with relevant guidelines and regulations. And all experimental protocols were approved by the Ethical Committee of Second Military Medical University and Shuguang Hospital. Patients provide informed consent authorizing the use of their personal information and tissues for research purposes.

### Ethics approval

This study was approved by the institution ethics review board for research involving human subjects at Second Military Medical University and Shuguang Hospital.

## Electronic supplementary material

Supplementary Information
