# Supplementary material for: SHP2 associates with nuclear localization of STAT3: significance in progression and prognosis of colorectal cancer
Source: Sci Rep. 2017 Dec 14;7:17597. doi: 10.1038/s41598-017-17604-7 (PMC5730547; doi:10.1038/s41598-017-17604-7)
Supplement: Supplementary file 1 — Supplementary Information [file 41598_2017_17604_MOESM1_ESM.pdf]

Supplementary Information

SHP2 associates with nuclear localization of STAT3: significance in progression and prognosis of colorectal cancer

Yan Huang[1]#, Jie Wang[1]#, Fuao Cao[2]#,Hailong Jiang[1], An Li[1], Jianzhong Li[1], Lei Qiu[1], Hao Shen[3], Wenjun Chang[3], Chuanxiang Zhou[4]*, Yamin Pan[5]*,Yiming Lu[1]*

1 Department of Biochemical Pharmacy, School of Pharmacy, Second Military Medical University, Shanghai 200433, China

2 Department of colorectal surgery, Changhai Hospital, Second Military Medical University, Shanghai 200433, China

3 Department of Environmental Hygiene, Second Military Medical University, Shanghai 200433, China

4 Department of Oral Pathology, Peking University School and Hospital of Stomatology, Beijing 100081, China

5 Department of Endoscopy, Shuguang Hospital, Shanghai University of Traditional Chinese Medicine. 201203, China.

# Co-first author

* Correspondence and requests should be addressed to Prof. Chuanxiang Zhou, Prof. Yamin Pan and Prof. Yiming Lu; Tel. /fax: +86 1082195221 (C. Zhou); +86 2120256691 (Y. Pan) and +86 2181871333 (Y. Lu); E-mail: zhoucx2008@126.com (C. Zhou), Panyamin2014@163.com (Y. Pan) and E-mail: bluesluyi@sina.com (Y. Lu).

Supplementary Figure 1-5

Supplementary Table 1-4

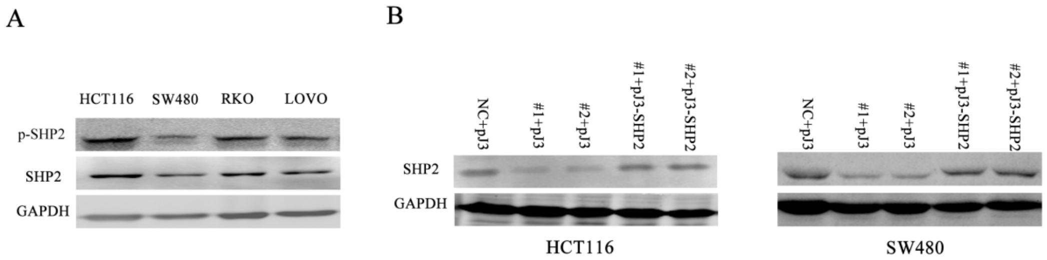
A
HCT116
SW480
RKO
LOVO
p-SHP2
SHP2
GAPDH
B
NC+pJ3
#1+pJ3
#2+pJ3
#1+pJ3-SHP2
#2+pJ3-SHP2
SHP2
GAPDH
HCT116
SW480

Fig. S1 (A) Expressions of SHP2 and p-SHP2 in CRC cells**.** (B) SHP2 overexpression rescued siRNA (#1 and #2) mediated SHP2 knockdown in HCT116 and SW480 cells.

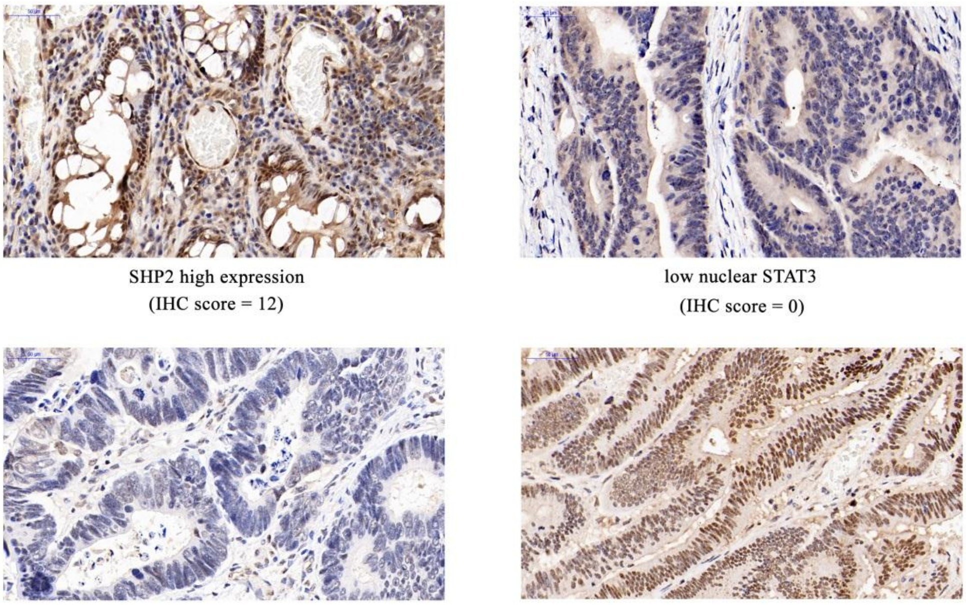

A

SHP2 high expression
(IHC score = 12)

low nuclear STAT3
(IHC score = 0)

B

SHP2 low expression
(IHC score = 0)

high nuclear STAT3
(IHC score = 12)

Fig. S2 SHP2 expression and STAT3 localization were analyzed by IHC (×200). (A) High levels of cytoplasmic SHP2 and low levels of nuclear STAT3 from the same site on the same patient. (B) Low levels of SHP2 and high levels of nuclear STAT3 from the same site on the same patient.

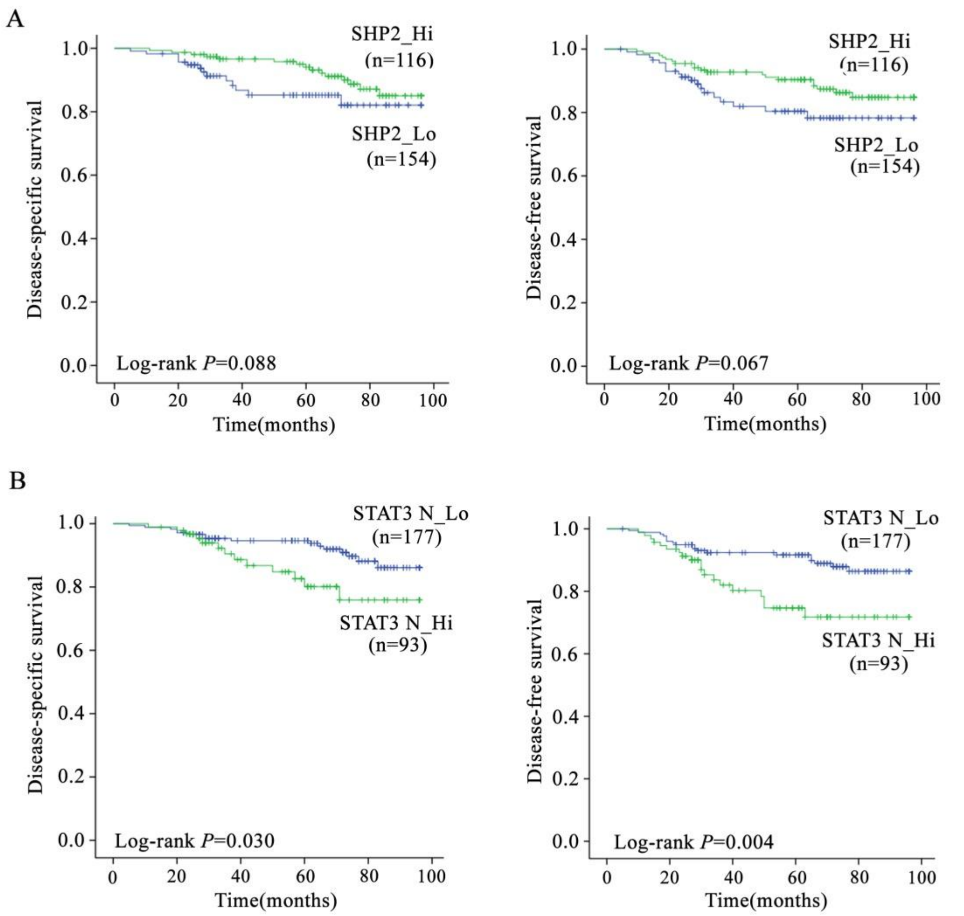
A
SHP2_Hi
(n=116)
SHP2_Lo
(n=154)
Disease-specific survival
1.0
0.8
0.6
0.4
0.2
0.0
Log-rank P=0.088
0
20
40
60
80
100
Time(months)
SHP2_Hi
(n=116)
SHP2_Lo
(n=154)
Disease-free survival
Log-rank P=0.067
Time(months)
B
STAT3 N_Lo
(n=177)
STAT3 N_Hi
(n=93)
Disease-specific survival
Log-rank P=0.030
Time(months)
STAT3 N_Lo
(n=177)
STAT3 N_Hi
(n=93)
Disease-free survival
Log-rank P=0.004
Time(months)

Fig. S3 Association between SHP2 or nuclear STAT3 and survival in patients with CRC. Kaplan-Meier analysis showed the levels of SHP2 could not be used to dichotomize 270 patients with CRC into different groups with distinct survival outcomes (A).Patients with low nuclear STAT3 present better DFS but not DSS than patients with high nuclear STAT3 (B).

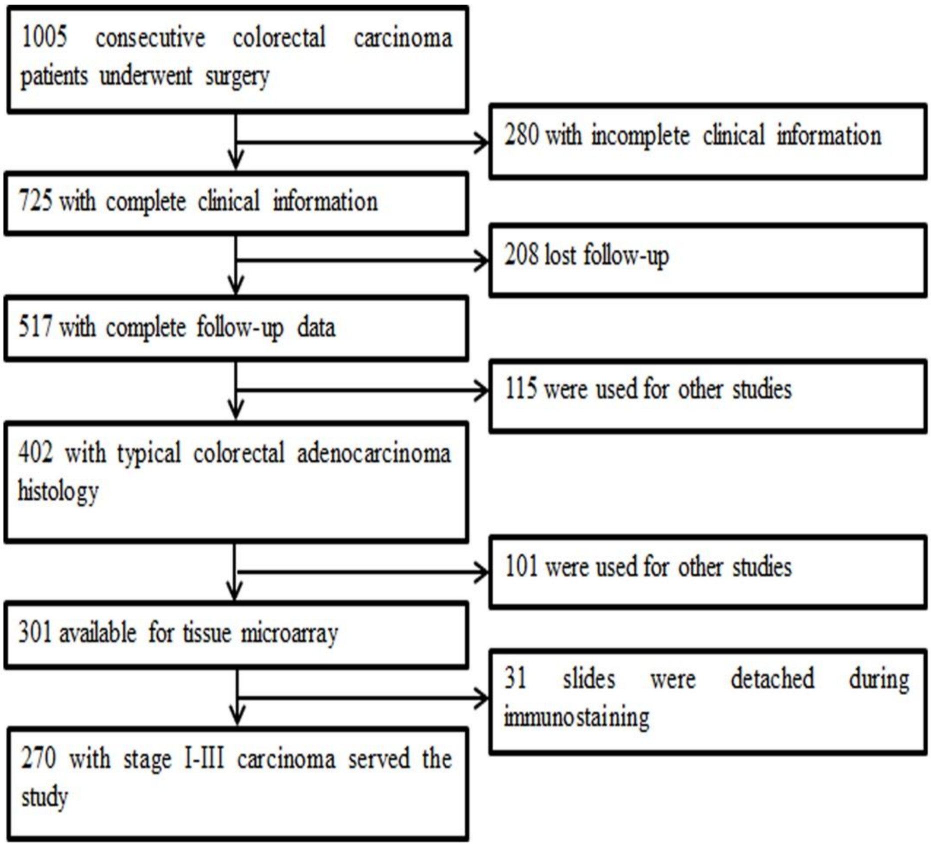
1005 consecutive colorectal carcinoma patients underwent surgery
280 with incomplete clinical information
725 with complete clinical information
208 lost follow-up
517 with complete follow-up data
115 were used for other studies
402 with typical colorectal adenocarcinoma histology
101 were used for other studies
301 available for tissue microarray
31 slides were detached during immunostaining
270 with stage I-III carcinoma served the study

Fig. S4 Flow diagram and selection criteria of study patients in this cohort.

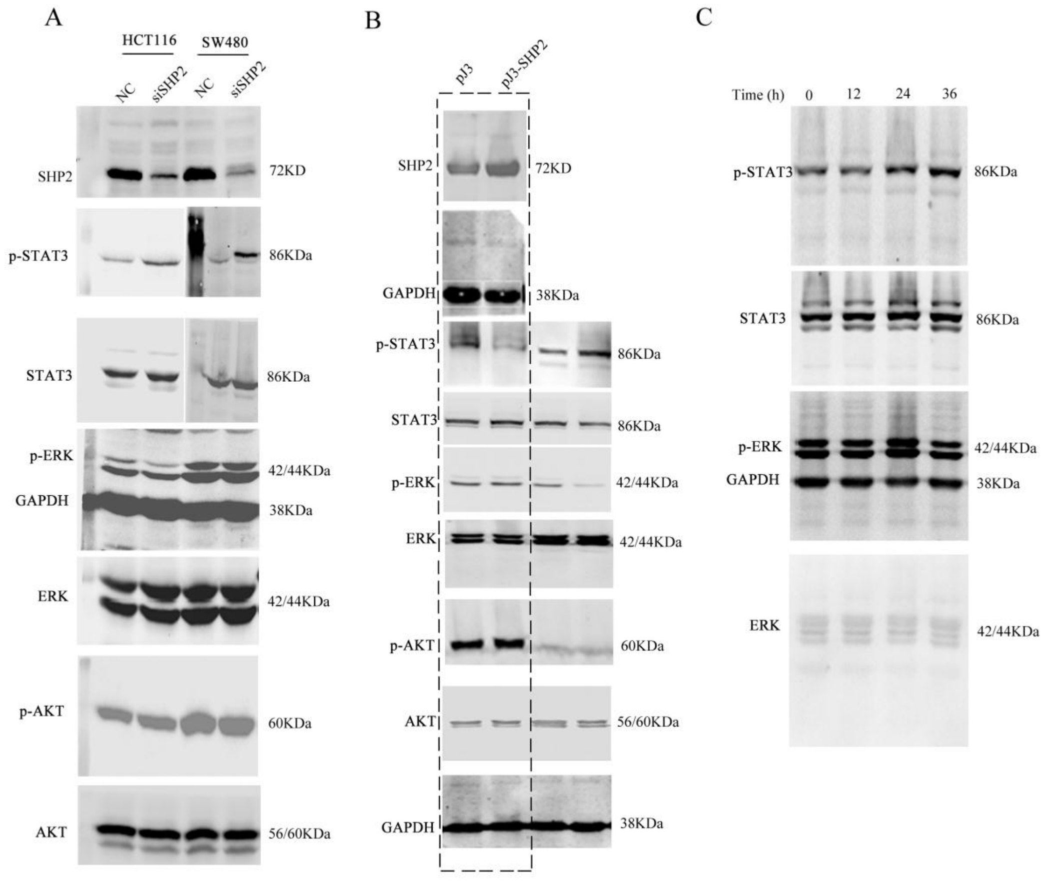
A
HCT116
SW480
NC
siSHP2
NC
siSHP2
SHP2
72KD
p-STAT3
86KDa
STAT3
86KDa
p-ERK
42/44KDa
GAPDH
38KDa
ERK
42/44KDa
p-AKT
60KDa
AKT
56/60KDa
B
pJ3
pJ3-SHP2
SHP2
72KD
GAPDH
38KDa
p-STAT3
86KDa
STAT3
86KDa
p-ERK
42/44KDa
ERK
42/44KDa
p-AKT
60KDa
AKT
56/60KDa
GAPDH
38KDa
C
Time (h)
0
12
24
36
p-STAT3
86KDa
STAT3
86KDa
p-ERK
42/44KDa
GAPDH
38KDa
ERK
42/44KDa

Fig. S5 Uncropped scans of western blot displayed in Fig. 2A, B, and C.

Table S1 Clinical parameters of CRC patients and the association with the combinations of cytoplasmic SHP2 and nuclear STAT3

	No. of cases	SHP2_Hi and STAT3_Lo	SHP2_Lo or STAT3 _Hi	*P*-value	SHP2_Lo and STAT3_Hi	SHP2_Hi or STAT3_Lo	*P*-value
Total	270	126	144		65	205	
Age (years)	270			0.475			0.296
Mean (SD)		61.24 (11.98)	62.30 (12.32)		61.55 (12.87)	61.89 (11.95)	
Sex (n (%))				0.511			0.44
Women	115 (42.6)	51 (40.5)	64 (44.4)		25 (38.5)	90 (43.9)	
Men	155 (57.4)	75(59.5)	80 (55.56)		40 (61.4)	115 (56.1)	
Disease location (n (%))				0.172			0.888
Colon	164 (60.7)	82 (65.1)	82 (56.9)		39 (60.0)	125 (61.0)	
Rectum	106 (39.3)	44 (34.9)	62 (43.1)		26 (40.0)	80 (39.0)	
TNM stage (n (%))				0.83			0.16
I	2 (0.7)	0 (0)	2 (1.4)		1 (1.5)	1 (0.5)	
II	177 (65.6)	85 (67.4)	92 (63.9)		37 (56.9)	140 (68.3)	
III	91 (33.7)	41 (32.6)	50 (34.7)		27 (41.6)	64 (31.2)	
T stage (n (%))				0.148			0.647
T1	2(0.7)	0(0)	2(1.4)		1(1.5)	1(0.5)	
T2	107(39.6)	71(56.3)	36(25.0)		17(26.2)	90(43.9)	
T3	91(33.7)	9(7.1)	82(56.9)		37(56.9)	54(26.3)	
T4	70(25.9)	46(36.5)	24(16.7)		10(15.4)	60(29.3)	
N stage (n (%))				0.427			0.738
N0	113(41.8)	63(50.0)	50(34.7)		26(40.0)	87(42.4)	
N1	124(45.9)	38(30.2)	86(59.7)		35(53.8)	89(43.4)	
N2	33(12.2)	25(19.8)	8(5.56)		4(6.2)	29(14.1)	
Differentiation grade (n (%))				0.746			0.776
Well	17 (6.3)	7 (5.1)	10 (7.5)		4 (6.2)	13 (6.3)	
Moderately	235 (87.1)	125 (91.9)	110 (82.1)		52 (80.0)	183 (89.3)	

Poorly	6 (2.2)	2 (1.5)	4 (3.0)		1 (1.5)	5 (2.4)	
Missing	12 (4.4)	2 (1.5)	10 (7.4)		8 (12.3)	4 (2.0)	
Adjuvant chemotherapy (n (%))				0.968			0.342
Yes	214 (79.3)	100 (79.4)	114 (79.1)		54 (83.1)	159 (77.6)	
No	56 (20.7)	26 (20.6)	30 (20.9)		11 (16.9)	46 (22.4)	
Serum CEA (ng/mL)				0.366			0.401
Median (rang)		3.52 (0-49.53)	3.83 (0-223.10)		4.08 (0-223.1)	3.61 (0-96.51)	
Serum CA19-9 (U/mL)	264			0.312			0.266
Median (rang)		13.30 (0-141.70)	11.69 (0-1000.0)		11.33 (0-1000.0)	12.49 (0-190.60)	
Missing	6						

Table S2.Cox regression analysis of DSS and DFS for clinical parameters, SHP2 and nuclear STAT3 expressions.

Variables	DSS				DFS			
	Univariate analysis		Multivariate analysis		Univariate analysis		Multivariate analysis	
	HR (95% CI)	*P*-value	HR (95% CI)	*P*-value	HR (95% CI)	*P*-value	HR (95% CI)	*P*-value
Nuclear STAT3 level(STAT3_Hi *vs* STAT3_Lo)	**2.139 (1.019-4.490)**	**0.044**	1.426 (0.611-3.330)	0.412	**2.402 (1.263-4.566)**	**0.008**	1.733 (0.827-3.631)	0.145
SHP2 level (SHP2_Hi *vs* SHP2_Lo)	0.519 (0.248-1.085)	0.081	0.518 (0.219-1.222)	0.133	0.541 (0.285-1.03)	0.061	0.605 (0.288-1.275)	0.187
Age (>50 *vs* ≤ 50)	1.388 (0.483-3.989)	0.543	1.306 (0.445-3.827)	0.627	0.713 (0.337-1.506)	0.375	0.692 (0.320-1.494)	0.348
TNM (III *vs* I+II)	1.551 (0.749-3.227)	0.24	1.433 (0.631-3.255)	0.390	1.62 (0.854-3.071)	0.139	1.379 (0.689-2.763)	0.364
Sex (women *vs* men)	1.892 (0.856-4.164)	0.113	0.459 (0.203-1.038)	0.062	**2.282 (1.107-4.704)**	**0.025**	**0.402 (0.191-0.849)**	**0.017**
Disease location (colon *vs*rectum)	1.032 (0.479-2.225)	0.936	0.925 (0.422-2.031)	0.846	0.984 (0.508-1.905)	0.961	0.886 (0.447-1.757)	0.729
Adjuvant chemotherapy (yes *vs* no)	1.185 (0.452-3.109)	0.730	1.002 (0.346-2.901)	0.997	2.162 (0.767-6.093)	0.145	1.775 (0.599-5.261)	0.301
Serum CEA (ng/mL) (≥5 *vs*<5)	**2.433 (1.160-5.101)**	**0.019**	**2.186 (1.000-4.777)**	**0.050**	1.841 (0.974-3.482)	0.60	1.859 (0.949-3.645)	0.071
Serum CA19-9 (U/mL) (≥37 *vs*<37)	1.869 (0.797-4.384)	0.151	1.597 (0.643-3.967)	0.313	1.273 (0.560-2.894)	0.564	1.113 (0.461-2.684)	0.812

Table S3. Agreement in the final evaluation of cytoplasmic SHP2 score in individual patients

Final evaluation of patient cytoplasmic SHP2 score		Observer #2											
		.00	1.00	3.00	4.00	5.00	6.00	7.00	8.00	9.00	10.00	11.00	12.00
Observer #1	.00	3	1	0	0	0	0	0	0	0	0	0	0
	3.00	0	0	3	0	0	0	0	0	0	0	0	0
	4.00	0	0	2	10	3	0	0	0	0	0	0	0
	5.00	0	0	0	1	3	2	0	0	0	0	0	0
	6.00	0	0	0	0	3	8	2	0	0	0	0	0
	7.00	0	0	0	0	0	2	4	1	0	0	0	0
	8.00	0	0	0	0	0	0	0	53	2	0	0	0
	9.00	0	0	0	0	0	0	0	1	12	0	0	0
	10.00	0	0	0	0	0	0	0	0	5	21	2	0
	11.00	0	0	0	0	0	0	0	0	0	2	27	2
	12.00	0	0	0	0	0	0	0	0	0	0	7	88

Table S4 Agreement in the final evaluation of nuclear STAT3 score in individual patients

Final evaluation of patient nuclear STAT3 score		Observer #2												
		.00	1.00	2.00	3.00	4.00	5.00	6.00	7.00	8.00	9.00	10.00	11.00	12.00
	.00	76	20	0	0	0	0	0	0	0	0	0	0	0
	1.00	0	58	0	0	0	0	0	0	0	0	0	0	0
	2.00	0	0	59	10	0	0	0	0	0	0	0	0	0
	3.00	0	0	0	18	0	0	0	0	0	0	0	0	0
	4.00	0	0	0	4	29	5	0	0	0	0	0	0	0
	5.00	0	0	0	0	0	15	0	0	0	0	0	0	0
Observer #1	6.00	0	0	0	0	0	2	25	2	0	0	0	0	0
	7.00	0	0	0	0	0	0	0	8	0	0	0	0	0
	8.00	0	0	0	0	0	0	0	0	9	3	0	0	0
	9.00	0	0	0	0	0	0	0	0	0	4	1	0	0
	10.00	0	0	0	0	0	0	0	0	0	0	9	0	0
	11.00	0	0	0	0	0	0	0	0	0	0	0	4	1
	12.00	0	0	0	0	0	0	0	0	0	0	0	0	10
